# Editorial: Antimicrobial Peptides: Molecular Design, Structure-Function Relationship, and Biosynthesis Optimization

**DOI:** 10.3389/fmicb.2022.888540

**Published:** 2022-04-11

**Authors:** Ya Hao, Jianhua Wang, Cesar de la Fuente-Nunez, Octavio Luiz Franco

**Affiliations:** ^1^Innovative Team of Antimicrobial Peptides and Alternatives to Antibiotics, Gene Engineering Laboratory, Feed Research Institute, Chinese Academy of Agricultural Sciences, Beijing, China; ^2^Key Laboratory of Feed Biotechnology, Ministry of Agriculture and Rural Affairs, Beijing, China; ^3^Machine Biology Group, Departments of Psychiatry and Microbiology, Institute for Biomedical Informatics, Institute for Translational Medicine and Therapeutics, Perelman School of Medicine, University of Pennsylvania, Philadelphia, PA, United States; ^4^Departments of Bioengineering and Chemical and Biomolecular Engineering, School of Engineering and Applied Science, University of Pennsylvania, Philadelphia, PA, United States; ^5^Penn Institute for Computational Science, University of Pennsylvania, Philadelphia, PA, United States; ^6^S-Inova Biotech, Universidade Católica Dom Bosco, Campo Grande, Brazil; ^7^Centro de Análises Proteômicas e Bioquímicas Programa de Pós-Graduação em Ciências Genômicas e Biotecnologia, Universidade Católica de Brasília, Brasília, Brazil

**Keywords:** antimicrobial peptides (AMPs), molecular design and modification, structure-function relationship, biosynthesis optimization, drug combination

The long-term use of antibiotics has accelerated the emergence of antibiotic-resistant bacteria (ARB). Annual global death due to antibiotic resistance is over 700,000 in 2014, it is predicted that a continued rise in resistance would lead to annual death rate of 10 million by 2050 (O'Neill, 2014; WHO, 2014). Antimicrobial peptides (AMPs) are a class of small molecules produced by numerous living organisms as part of their host innate immune response to infection (Loose et al., 2006; Torres and de la Fuente-Nunez, 2019; Cesaro et al., 2022). AMPs evolution in insects and other species have demonstrated the ability of these molecules to eliminate invading pathogens and thus have generated a great excitement at the prospect of developing AMPs as alternatives to antibiotics (ATAs) for years (Czaplewski et al., 2016; Magana et al., 2020).

The field of AMP research started in the 1980s owing to the discoveries of insect cecropins by Hans Boman, human α-defensins by Robert Lehrer, and magainins by Michael Zasloff (Wang et al., 2016). Over 3,300 kinds of AMPs have now been found in a wide range of biological sources, ranging from microbes, plants, to animals (Torres et al., 2022). These peptides may possess optimal properties for further drug development, including their ability to permeabilize and disrupt the bacterial membrane, capability of regulating the immune system and also broad-spectrum antibiofilm activity, and reduced propensity to select for bacterial resistance (de la Fuente-Núñez et al., 2012, 2016; Yang et al., 2019; Wang et al., 2022). These advantages of AMPs over conventional antibiotics have attracted attention despite that their use have primarily been limited to topical infections due to their relative narrow druggability and lack of special unique protocols to assess pharmacodynamics (Liu et al., 2021a; Ma et al., 2021; Dos Santos-Silva et al.). The highlights of 21 papers in this topic will be briefly presented and reviewed as follows.

## Discovering New Natural AMPs

Natural product and their special functional structures or domains have traditionally played a significant role in drug discovery and development (Newman and Cragg, 2012). Plant AMPs are rich in diversity and have been reported to have antimicrobial activity against infections caused by pathogens (Dos Santos-Silva et al.). Lee et al. isolated a novel peptide PN5 from pine needles of *Pinus densiflora*. Sieb. et Zucc, exhibiting a strong antimicrobial activity against foodborne bacteria, and no detectable cytotoxicity. In fact, animals, plants, and microbiota harbor a large number of bacteria that they compete for nutrients and space and exchange biomolecules (Tobias et al., 2017). *In vivo* competition and interspecies exchange involve the synthesis and continuous evolution of many previously unknown AMPs. Ngashangva et al. isolated a new AMP from a bacterial endophyte derived from the medicinal plant *Millettia achycarpa* Benth and analyzed its biosynthetic gene cluster by collective analysis of both genomic and proteomic data. In addition, Brevibacillin 2V, a novel lipo-tridecapeptide with a strong antimicrobial activity against antibiotic-resistant *Staphylococcus aureus* ATCC15975 (MRSA) was reported to show much lower hemolytic activity and cytotoxicity toward eukaryotic cells than previously reported non-ribosomally produced peptides from the lipo-tridecapeptide family, making it a promising candidate for new peptide antibiotic development (Zhao et al.). However, AMPs' toxicity, instability, low yield obtained by recombinant expression, and high cost of chemical synthesis have hindered their application and translation into the clinic. Recent advances in data mining, artificial intelligence (Porto et al., 2018b; de la Fuente-Nunez, 2019; Torres et al., 2022), synthetic biology, chemical biology, and interdisciplinary tools are greatly pushing the pace of new discoveries and solutions to overcome these drawbacks.

## Molecular Design of AMPs

Advances in methodological design are necessary to discover and advance new AMPs. *De novo* design involving the latest theoretical knowledge is being applied to determine and screen quickly an economically feasible strategy for short candidate AMPs sequences with basic antibacterial ability and high selectivity (Wang et al.). In recent years, with the development of big data and artificial intelligence, database screening and mining technology have become an exciting tool for drug discovery. Based on the AMP data pool APD, new target peptides with specific potent properties were designed and candidates with the most likely target activity based on key parameters were efficiently identified (Mishra and Wang, 2012; Magana et al., 2020). Using this method, Bobde et al. screened eight novel AMPs, termed PHNX peptides, against Gram-negative bacteria. Advances in the development of computational tools have greatly facilitated the discovery of novel peptide-based drugs. For example, a generative model (Porto et al., 2018a) was used to yield synthetic peptides with anti-infective efficacy in mouse models (Porto et al., 2018b; Torres et al., 2021). Recently, an algorithmic approach was used to explore the human body as a source of peptide antibiotics (Torres et al., 2022). In this issue, Dean et al. built an improved automated semi-supervised approach termed PepVAE for generating promising new sequences using a variational autoencoder.

## Molecular Modification of AMPs

Recently, the design and modification of AMPs have been extensively used for drug development purposes. Molecular modification is no longer limited to site substitution of select residues (Han et al.; Zhao et al.). An increasing number of additional tools have been developed such as chemical modification, cyclization, insertion of large hydrophobic side-chains (Liu et al., 2021a), chimera (Yu et al.) and polymerization, as well as their combination or cross (Hao et al., 2017; Li et al., 2018a,b; Wang et al., 2020). The conformational and physicochemical properties of AMPs play important roles in determining antibacterial activity, toxicity and bioavailability. The distribution of positive charge and hydrophobic amino acid within the helical wheel seems to be the key factors determining the antibacterial activity of AMPs, and their selectivity can be effectively improved by adjusting amphiphilicity (Luo et al.). Steigenberger et al. designed a membrane-permeabilizing lipopeptide with different chain-length and found its antimicrobial specificity depending on membrane difference of the target bacteria. These results reveal the importance and weight of taking into account bacterial membrane composition when designing AMPs.

## Nanotechnology Applied to AMPs

Most AMPs, if administered systemically into the body, may cause side effects or be degraded through multiple proteolytic cleavage. A gradual decrease, distribution, and slow accumulation of AMPs level released *in vivo* may lead to the induction of bacterial resistance at sub-inhibitory concentrations (Tan et al., 2021). To overcome these potential issues, nanotechnology has entered the realm of AMP application, and nanocarriers can be used as delivery systems in order to enhance therapeutic effects and minimize above undesirable side-effects (Magana et al., 2020). Nanocarriers can help improve the pharmacokinetic/pharmacodynamic profiles of AMPs, extending shelf life and half-life, stability and bioavailability. With the development of nanotechnology, a variety of peptide-based antibacterial nanomaterials have been produced as metal nanoparticles or carbon nanotubes conjugated AMPs, polymeric material nanosystem containing AMPs, and self-assembled AMPs (Yang et al.). More and better exciting new results in this field are expected in the near future.

## Recombinant Expression of AMPs

Considering the high cost of new drug development and feasible competition with existing antibiotics, mass production of AMPs at low cost is necessary and essential for deployment of these agents in the population. AMPs are produced at very low levels in living organisms, and their extraction is difficult, inefficient, expensive, and time consuming. Recombinant expression of peptides in DNA holds promise to enable large-scale production of AMPs at an affordable cost. Wang' team (Zhang et al., 2011, 2014; Cao et al., 2015; Li et al., 2017, 2020; Yang et al., 2019; Liu et al., 2020, 2021b; Shen et al.) and others (Cao et al., 2018) have been working in this area for decades, obtaining exciting peptide yields higher than 1 g (target peptide)/L(ferment supernatant) secreted from yeast cells (Zasloff, 2016; Sampaio de Oliveira et al., 2020).

## Synergism and Combination of AMPs Administration

AMPs have been shown to potentiate the activity of conventional antibiotics to target pathogens (Reffuveille et al., 2014; de la Fuente-Núñez et al., 2015). Synergism can enhance the antimicrobial activity of peptides (Zhang et al., 2014; Zhao et al., 2019; Ma et al., 2021) while reducing the amount of drug dosage needed to kill bacteria both by the peptide and antibiotic, markedly reducing the risk of ARB development. The specific molecular mechanisms underlying the synergistic effects between AMPs and other antibiotics are still unclear, although it has been hypothesized that the ability of peptides to penetrate into bacterial cells is a key contributor (Reffuveille et al., 2014). Wu et al. attempted to uncover more details into this by examining the effects of bulky non-natural amino acid end tagging strategy of short AMPs on their combination with conventional antibiotics against resistant bacteria.

## Conclusion

**Figure 1:**
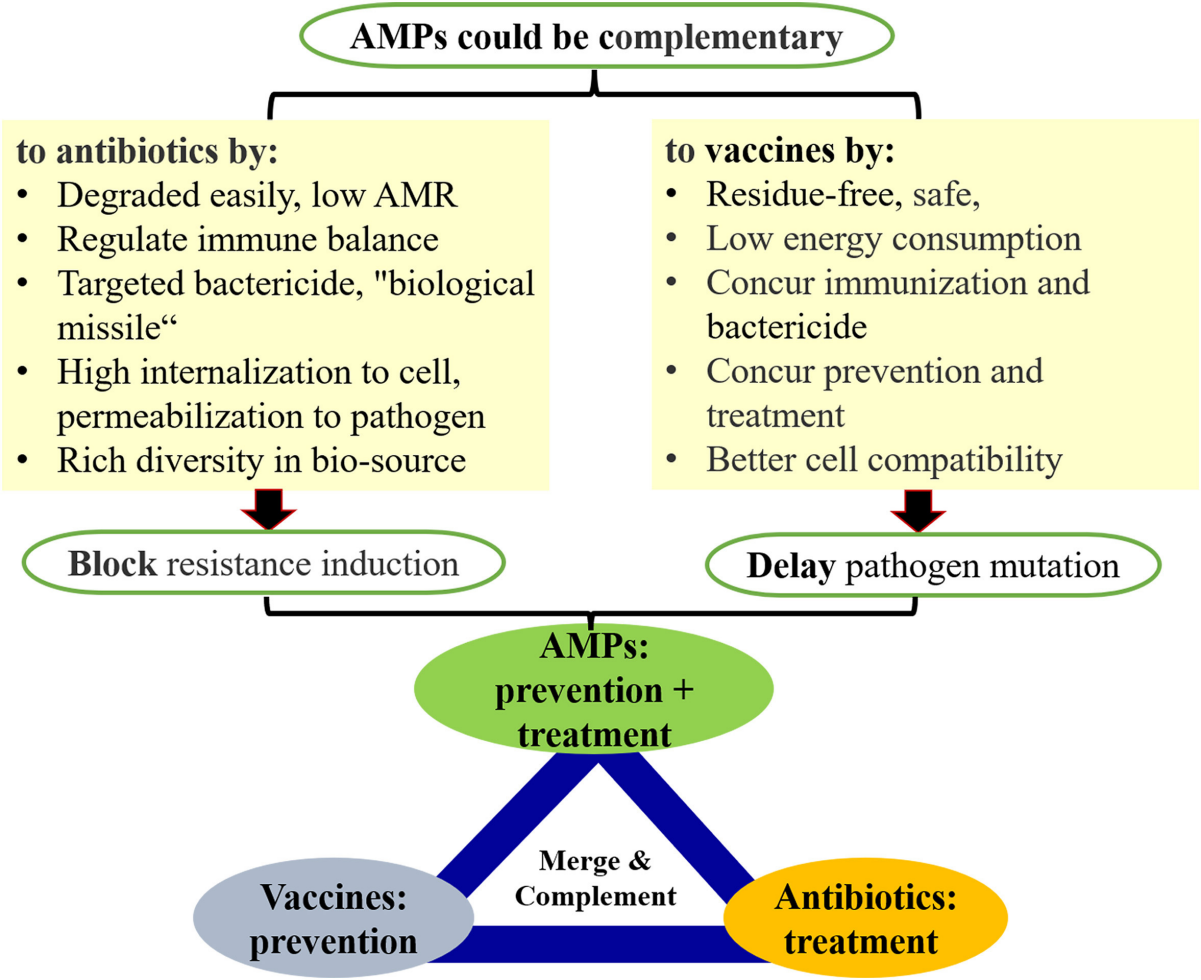
An iron triangle of health protection from AMPs, antibiotics and vaccines for maintaining a reasonable equilibrium among pathogens, antimicrobials and drug resistances is important and essential for one health management at safety level (Hao et al., 2017; Wang et al., 2018, 2022; Liu et al., 2021a; Zheng et al., 2021).

AMPs constitute the first line of innate defense against infection. These molecules are attractive drug candidates due to their multifactorial mechanism of action, low propensity to select for bacterial resistance, among other things (Hao et al., 2017; Wang et al., 2018, 2022; Liu et al., 2021a; Zheng et al., 2021). A reasonable equilibrium between antimicrobials and infectious pathogens is critical in order to control high resistance and high variation among pathogens, and can potentially be achieved by the appropriate use of AMPs, antibiotic and vaccines, as the iron triangle theory summarized in Figure 1. Future work should focus on determining the sequence requirements underlying for special unique PK/PD profiles of peptides, along with formulation, and ADME-Toxicity studies (Andes et al., 2009; Xiong et al., 2011; Magana et al., 2020) in order to translate AMPs into the clinic as soon as possible.

## Author Contributions

The first draft text of this editorial was written by Ph.D. student YH as assistant of JW with his guide and direction. All authors listed have made substantial, direct, and intellectual contribution to the work and approved it for publication.

## Funding

JW was supported by the National Natural Science Foundation of China (Grant no. 31872393), the Agricultural Science and Technology Innovation Program (ASTIP) in CAAS (CAAS-ASTIP-2017-FRI-02), and its key projects (CAAS-ZDXT2018008 and CAAS-ZDRW202111). OF was also supported by CAPES, CNPq, FAPDF, and FUNDECT.

## Conflict of Interest

The authors declare that the research was conducted in the absence of any commercial or financial relationships that could be construed as a potential conflict of interest.

## Publisher's Note

All claims expressed in this article are solely those of the authors and do not necessarily represent those of their affiliated organizations, or those of the publisher, the editors and the reviewers. Any product that may be evaluated in this article, or claim that may be made by its manufacturer, is not guaranteed or endorsed by the publisher.
